# Study on synergistic system of energy-absorbing yielding anti-impact supporting structure and surrounding rock

**DOI:** 10.1038/s41598-021-04631-8

**Published:** 2022-01-12

**Authors:** Yimin Song, He Ren, Hailiang Xu, Xu Guo, Zheng Chen, Dong An

**Affiliations:** School of Civil Engineering, North China University of Technology, Beijing, China

**Keywords:** Civil engineering, Mineralogy

## Abstract

Through the improvement of supporting structure and the utilization of the interaction between surrounding rock and supporting structure, the synergistic system of energy-absorbing yielding anti-impact supporting structure and surrounding rock is established. The process of energy absorption device, energy-absorbing yielding anti-impact supporting structure and synergistic system under impact is simulated to analyze the properties of them. The following conclusions could be drawn. The deformation and yielding process under compression of energy absorption device is divided into five stages. Compared with the traditional supporting structure, the energy-absorbing yielding anti-impact supporting structure has the reaction force with lower value and smaller fluctuation range before the deformation of the energy absorption device reaches the third ascending section. The synergy between surrounding rock and supporting structure plays an important role in roadway support. Compared with the supporting structure without surrounding rock, the reaction force of the supporting structure in the synergistic system is lower, and a stationary stage is added in the early stage of the reaction force curve.

## Introduction

With the increasing of mining depth^1–4^, great changes have taken place in the mechanical environment of mining^5,6^. In-situ stress increases nonlinearly with depth, which makes dynamic disasters such as rock burst more serious. When coal mine rock burst occurs, huge energy released will destroy surrounding rock of mining roadway and cause casualties^7–11^.

In recent years, much effective research on exploring the mechanism of rock burst has been carried out around the world. Vardoulakis^12^ analyzed the surface instability of a semi-infinite layer of cohesive-frictional material under plane-strain uniaxial compression. Chen et al.^13^ presented a double rock sample model for studying the rock burst mechanism. Tang et al.^14^ presents a numerical approach for the simulation of damage initiation and propagation causing seismic energy release during unstable failure of brittle rock. Li et al.^15^ studied rock burst within a deeply buried tunnel by numerical methods. Dou et al.^16^ proposed the principle of the rock burst induced by the combination of dynamic and static stresses.

So far, there are two basic directions to prevent rock burst, one is to reduce the impact energy brought by rock burst, and the other is to increase the energy absorption capacity of the whole supporting system. However, the initial energy caused by rock burst is still uncontrollable. And the supporting system is able to take over only a certain part of the energy resulting from the classification of energy balance^17^. Therefore, it is considered to improve the energy absorption capacity of supporting system, so as to reduce the impact energy acting on surrounding rock. Based on the above features, principles of rock burst prevention supporting system design are put forward by Pan et al.^18^. Besides, Fu et al.^19^ studied the failure mechanism of rock burst and demonstrated the buffering effect of high impact toughness anchor on impact energy. Gao et al.^20^ proposed the anchor cable active support-hydraulic lifting shed reduction span strong support-soft structure energy absorption technology. The relationship between the deformation process, load–displacement curve, and plastic strain of the original prefolded energy absorbing device is studied by Xu et al.^21^.

In this study, through the improvement of supporting structure and the utilization of the interaction between surrounding rock and supporting structure, the synergistic system of energy-absorbing yielding anti-impact supporting structure and surrounding rock is established. The process of energy absorption device, energy-absorbing yielding anti-impact supporting structure and synergistic system under impact is simulated to analyze the properties of them. The results prove that the system has better performance, and we hope that these findings can provide some help for the development of rock burst prevention and control in mining roadways.

## Project overview

### Background of theory and application

In view of the limitations of the existing mining roadway support, six principles of rock burst prevention support design are proposed by Yishan et al.^18^, namely, the design with variable abdicating resistance, the design with variable abdicating displacement, the design with variable abdicating stiffness, the design with variable abdicating frequency, the design with variable abdicating velocity and the design with variable abdicating energy.

**Figure 1:**
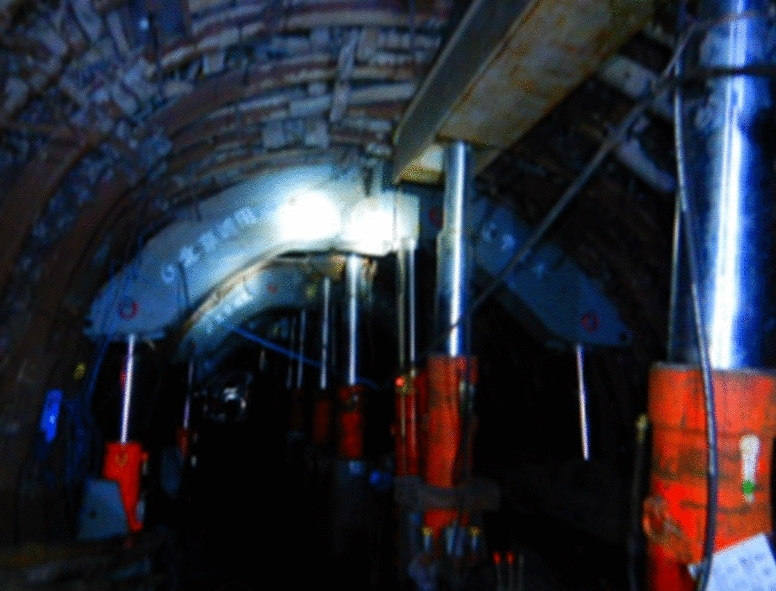
Field application of the supporting structure.

Based on the above principles, the energy-absorbing yielding anti-impact supporting structure was proposed and has been put into application in Gengcun Coal Mine which is located in Sanmenxia City, Henan Province, China. Figure 1 shows the field application of the supporting structure. On December 22, 2015, a rock burst with a magnitude of M_L_ 2.7 occurred at the Gengcun coal mine, releasing 2.3 × 10^6^ J of energy at the working face, destroying 160 m of the roadway and causing two deaths. On June 10, 2017, after the supporting system was installed, a mine seismic event with a release energy greater than 1 × 10^7^ J occurred, with the roadway intact and no casualties.

### Synergistic system

**Figure 2:**
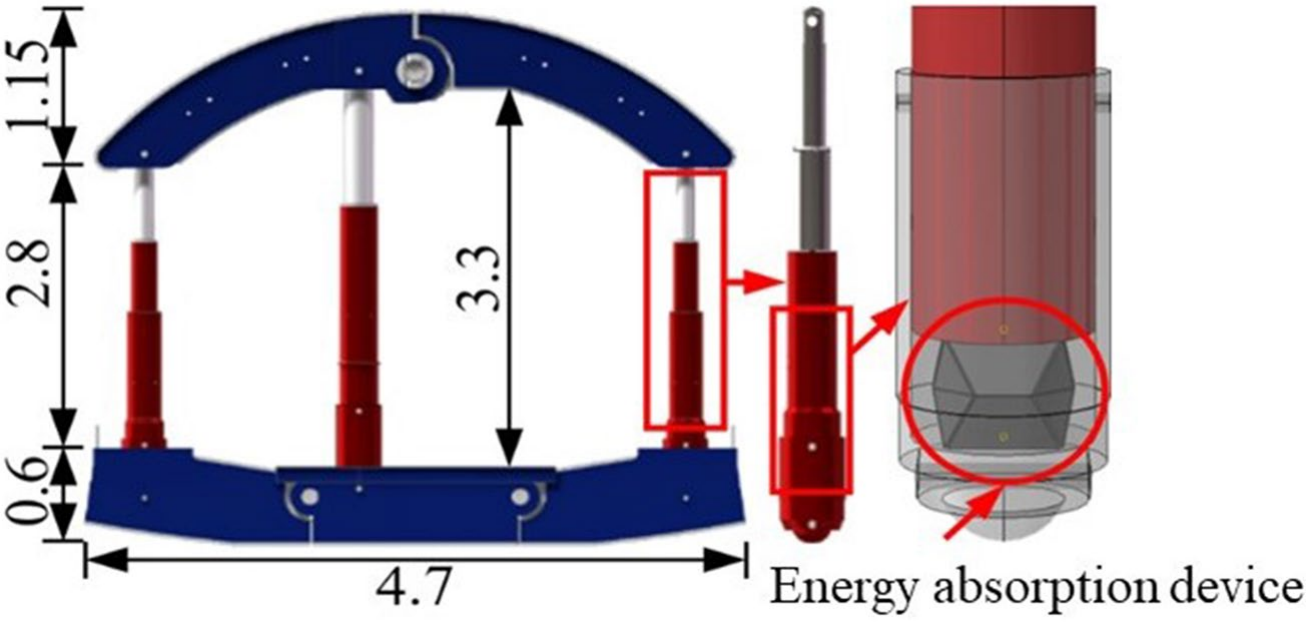
Energy-absorbing yielding anti-impact supporting structure.

**Figure 3:**
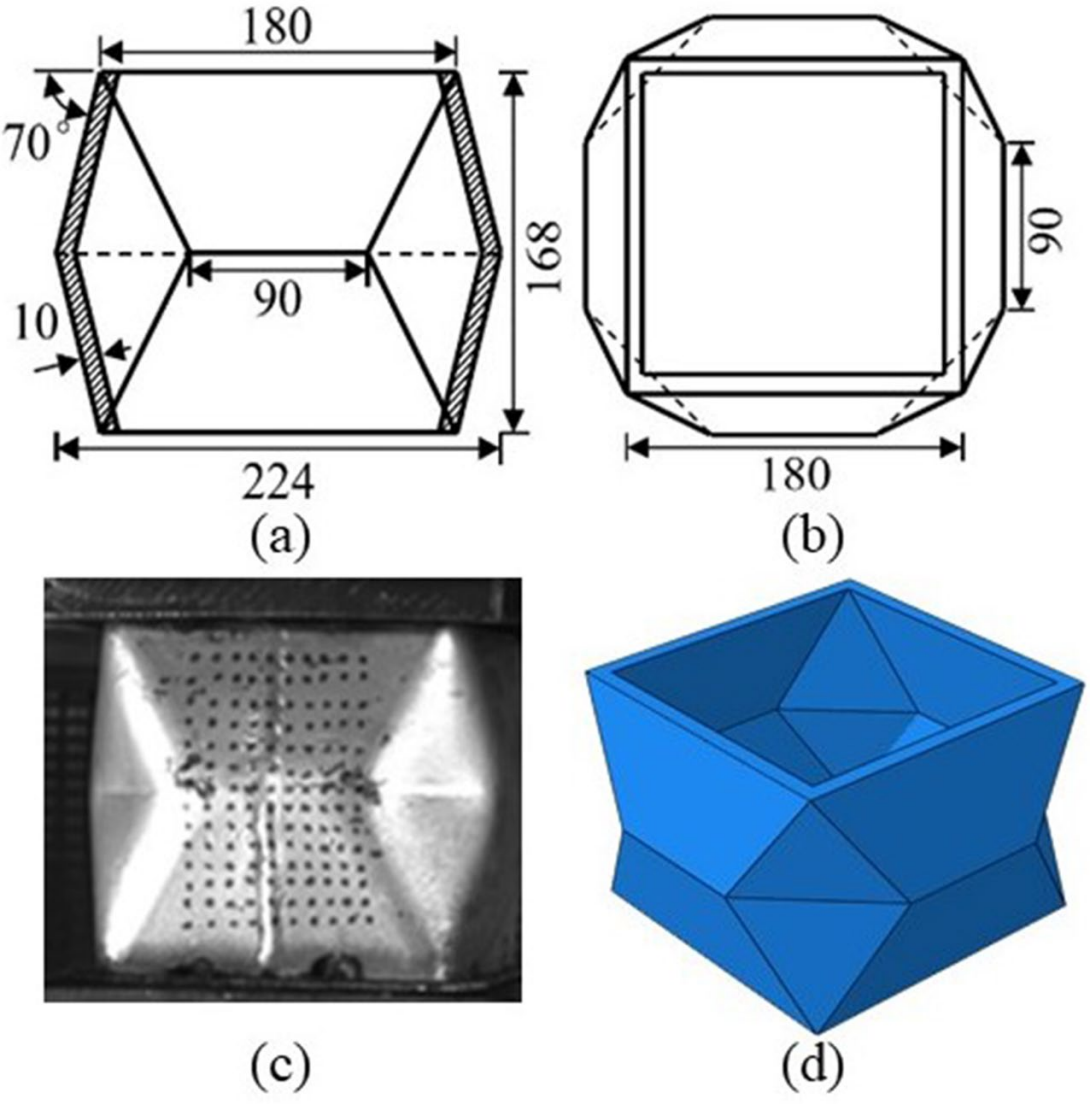
Energy absorption device: (**a**) front view of energy absorption device. (**b**) Top view of energy absorption device. (**c**) Picture of energy absorption device. (**d**) Three-dimensional drawing of energy absorption device.

The synergistic system consists of energy-absorbing supporting structure and surrounding rock. The advancement of the system lies in the improvement of supporting structure and the utilization of the interaction between surrounding rock and supporting structure. Figure 2 shows the structural composition of energy-absorbing yielding anti-impact supporting structure. The structure has eight basic components: left top beam, right top beam, middle column, left column, right column, left bottom beam and right bottom beam. Among them, the three columns are double telescopic hydraulic prop, which is the main stress system of the whole hydraulic support. In order to strengthen the integrity of the structure, the two parts of the top beam and the three parts of the bottom beam are connected by limiting hinges. The energy absorption devices are arranged at the bottoms of the three columns, which can realize the yielding of the whole supporting structure and prolong the impact time through compression deformation. It is designed from a thin-walled metal square tube, which is made of steel with a density of 7980 kg/m^3^, an elastic modulus of 210 GPa, a Poisson’s ratio of 0.3, a yield strength of 890 MPa and an ultimate strength of 1050 MPa. The size of it is shown in Fig. 3.

### ABAQUS

**Figure 4:**
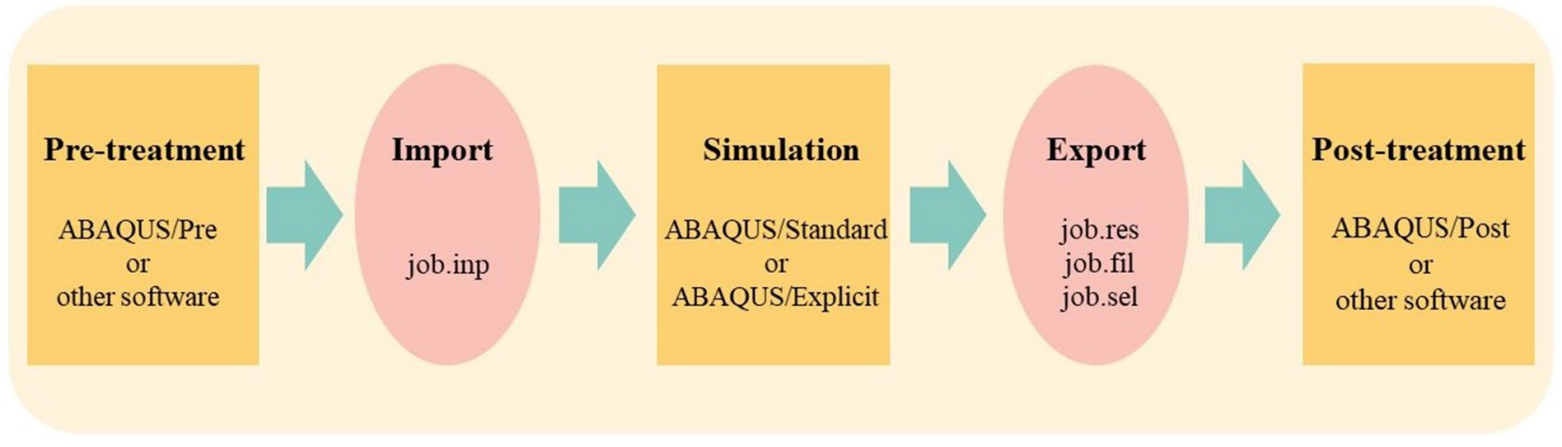
Analysis process of ABAQUS.

Numerical simulation based on ABAQUS is taken as the main research method in this paper. Abaqus is part of SIMULIA family of codes which is one of the most advanced large-scale finite element calculation and analysis software in the world. The analysis process of it is shown in Fig. 4. With the advantages of convenient use, nonlinear analysis function, rich unit library and material model library, and good openness, it has been widely used in industry and research in various countries.

## Analysis on the properties of energy absorption device

Experiment and numerical simulation are adopted in this section to explore the properties of energy absorption device, among which the loading method of the experiment is quasi-static compression.

In the numerical simulation, the material property of energy absorbing device is set to ideal elasto-plasticity. A downward-displacement rigid plate is set at the top of the device to simulate the compression effect of the press machine, and the displacement speed is consistent with the experiment. A fixed rigid plate is set at the bottom of the energy-absorbing device. The device and the plate are set in frictional contact, and the friction coefficient is set to 0.45.

**Figure 5:**
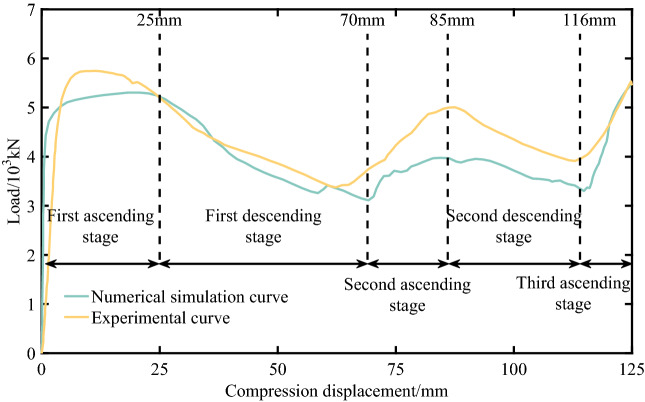
Load-compression displacement curve of numerical simulation and experiment.

**Figure 6:**
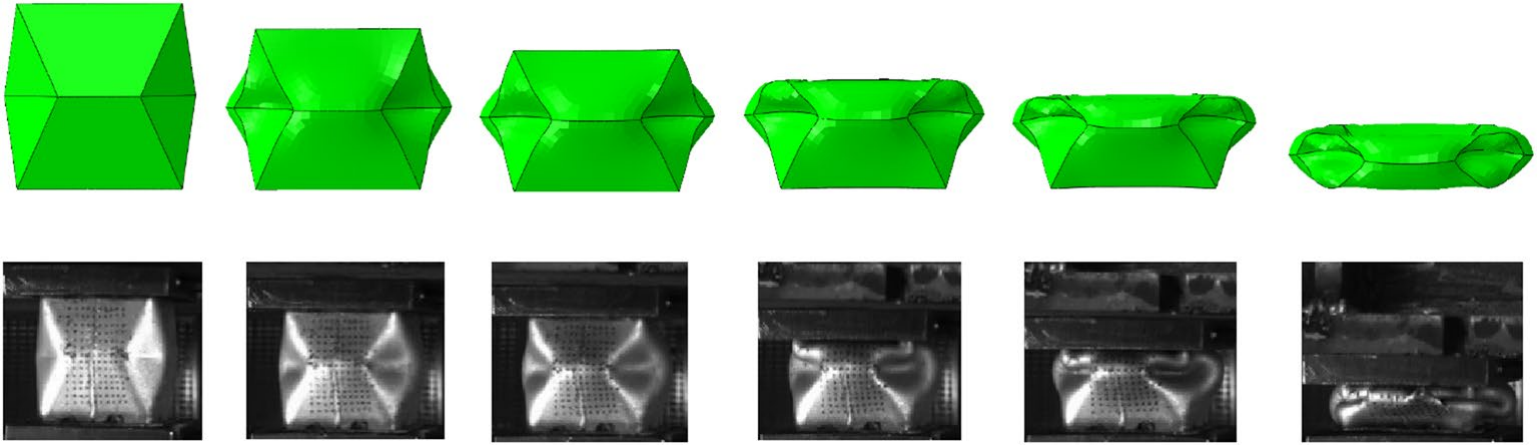
Crushing process diagram of numerical simulation and experiment.

The simulation and experimental results of the energy absorption device are shown in Figs. 5 and 6. It can be seen that the numerical simulation results are in good agreement with the experimental results. According to the load–displacement curve, the whole process of deformation and yielding is divided into five stages. The first ascending stage is the part where the compression displacement is less than 25 mm, and the peak load in this stage is 5401 kN. The first descending stage is within the range of 25–70 mm, and the peak load drops to 3116 kN. The second ascending stage is within the range of 70–85 mm, and the peak load increases to 4029 kN. The second descending stage is within the range of 85–116 mm, and the peak load drops to 3069 kN. After the compression displacement is greater than 116 mm, it is the third ascending stage. At this time, the energy-absorption device has been completely compressed and continues to compress, and the energy-absorption device no longer absorbs energy, that is, the energy-absorption device fails.

## Analysis on the properties of energy-absorbing yielding anti-impact supporting structure

### Energy-absorbing yielding anti-impact supporting structure model

**Figure 7:**
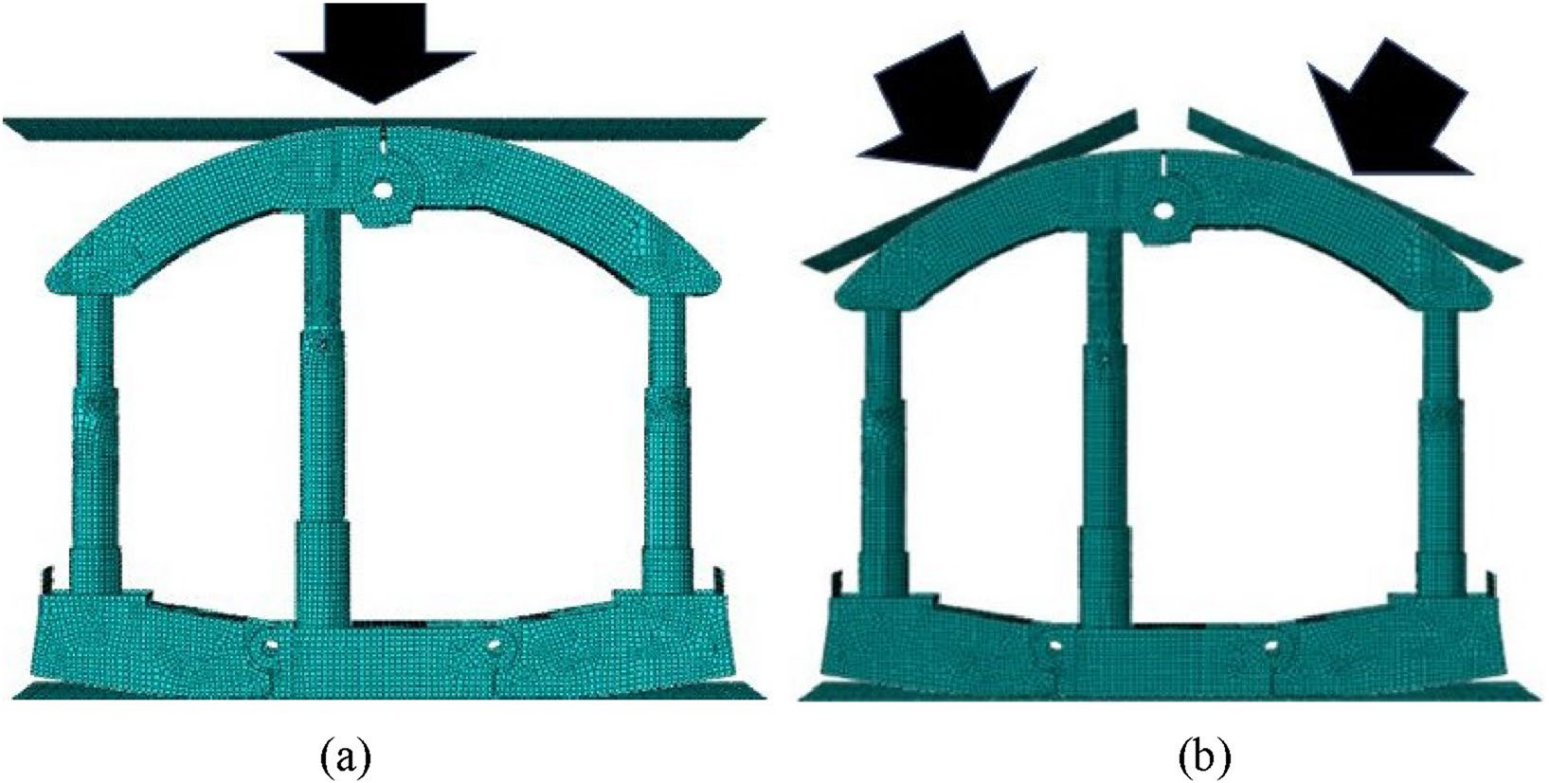
Energy-absorbing yielding anti-impact supporting structure under two working conditions: (**a**) Top impact condition. (**b**) Side impact condition.

The model of energy-absorbing yielding anti-impact supporting structure is presented in Fig. 7. The 7-node double-bending universal shell element S4 is used to mesh the model, with a mesh size of 35 mm and a total of 27,659 elements. The material used in the structure is Q550, with elastic modulus of 210 GPa, Poisson’s ratio of 0.3 and yield strength of 550 MPa. The contact between parts is tangentially contacted by penalty function, and the normal direction is set as hard contact. The model of traditional supporting structure is the same as before, and the position of the energy absorption device is changed to rigid connection. S4R universal shell element is used to mesh the model, with a mesh size of 30 mm and a total of 28,556 elements. The area where stress concentration may occur in the model is refined. In the whole process, the ideal elasto-plasticity is set as the material property, without considering the hardening characteristics. Two working conditions are adopted in this section, which are top impact and side impact. Simulation of impact by setting the impact velocity on the rigid plate. The impact energy is calculated as shown in Eq. (1).

1 $$ E = mv^{{2}} /{2}. $$

In Eq. (1), *m* is the weight of rigid plate, *v* is the impact velocity, *E* is the impact energy.

By comparing the reaction force and its frequency spectrum of energy-absorbing yielding anti-impact supporting structure under different working conditions with traditional supporting structure, the performance of energy absorbing structure is analyzed.

### Results of energy-absorbing yielding anti-impact supporting structure model

**Figure 8:**
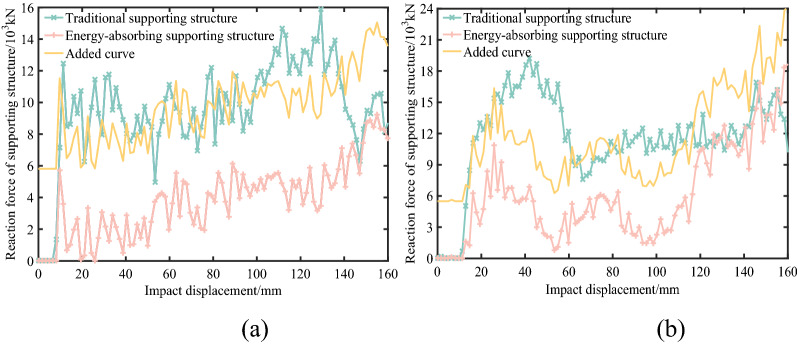
Reaction force of supporting structure: (**a**) under top impact condition. (**b**) Under side impact condition.

Figure 8 presents the reaction force curves of supporting structure under different working conditions. In the early stage of impact action, that is, before the impact deformation is about 10 mm, both supporting structures have great supporting ability. Before the impact displacement reaches 120 mm, the overall fluctuation trend of reaction force between two supporting structures is basically the same. In order to observe this phenomenon more intuitively, a curve shifted upward according to the difference of average reaction force between them is added in Fig. 8. However, in terms of numerical value, there is a huge difference between the two kinds of structures. Under top impact, the average reaction force of traditional structure is 9629.8 kN, and that of energy-absorbing structure is 3818.9 kN, which is only 40% of the former. Under side impact, the average reaction force of traditional structure is 11,673.5 kN, and that of energy-absorbing structure is 6186.7 kN, which is only 53% of the former. Therefore, before the impact displacement reaches 120 mm, the energy absorption device can effectively reduce the reaction force on structure under impact. That is, disturbance to surrounding rock of mining roadway caused by impact is reduced. When the impact displacement reaches 120 mm, the reaction force of the energy-absorbing supporting structure gradually increases. The reason is that the deformation state of the energy absorption device enters the third rising stage, and the function of it gradually fails. And after the impact displacement reaches 140 mm, the energy absorption device is completely ineffective. At this time, the reaction forces of the two supporting structures are consistent. The whole energy absorption process of the energy absorption device lasts about 0.014 s.

**Figure 9:**
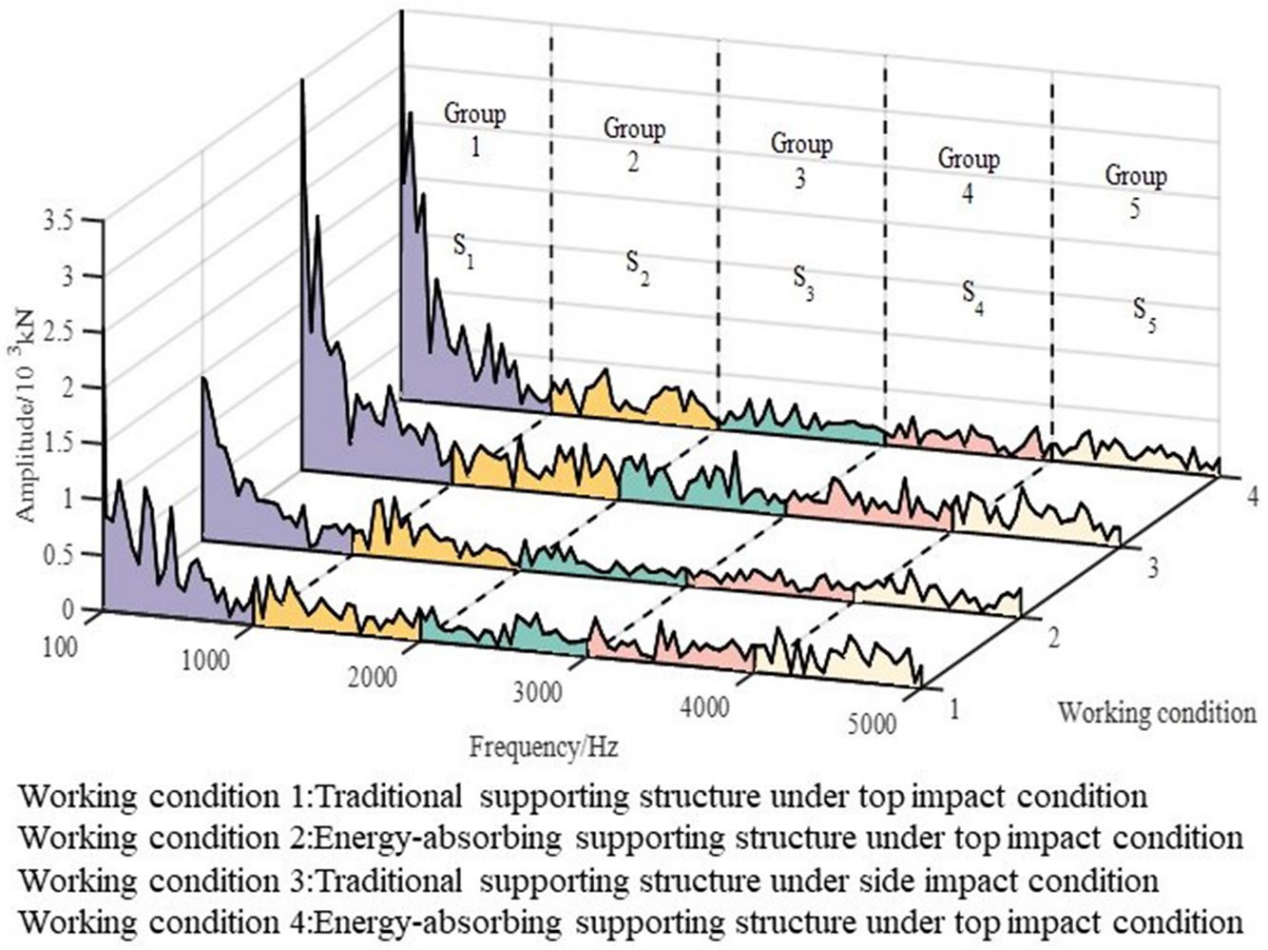
Frequency spectrum characteristic curve of reaction force.

The frequency spectrum characteristic curve of reaction force is obtained by Fourier transform, as shown in Fig. 9. The Fourier transform formula is given in Eq. (2).

2 $${X}_{k}=\sum_{n=0}^{N-1} {x}_{n}{e}^{\frac{-i2\pi kn}{N}},k=\mathrm{0,1},2,\dots ,N-1.$$

In Eq. (2), $N$ is sample number, ${x}_{n}$ is time domain sample, ${X}_{k}$ is frequency domain sample.

The whole impact process lasts only about 0.02 s, so the low vibration frequency is ignored in the analysis process. In order to quantitatively compare the fluctuation differences of various working conditions, the frequency range is divided into five groups, which are 100–1000 Hz, 1000–2000 Hz, 2000–3000 Hz, 3000–4000 Hz and 4000–5000 Hz. The time history points of discrete spectrum data in each working condition are fitted, and the area and its proportion in each fundamental frequency group are calculated. Then, the fundamental frequency distribution of reaction force is analyzed, and the fluctuation characteristics under different working conditions are compared. The calculation results are shown in Table 1. Compared with the traditional structure, the frequency distribution of the first two groups of energy-absorbing structure increased, while the frequency distribution of the third, fourth and fifth groups of that decreased. The results shows that the reaction force fluctuation range of the energy-absorbing supporting structure under impact is smaller. That is the energy absorption device can effectively slow down the fluctuation of supporting reaction force under impact. Furthermore, the adverse effect of secondary impact on surrounding rock around the mining roadway caused by extremely rapid fluctuation of reaction force under the impact action of the supporting structure can be reduced.

**Table 1:** Calculation results of fundamental frequency group distribution.

Working condition	Fundamental frequency group	S1	S2	S3	S4	S5
Traditional supporting structure under top impact	Area/(× 10^3^ kN·Hz)	482.62	215.15	164.83	171.67	228.67
	Area ratio/%	38.21	17.04	13.05	13.59	18.11
Energy absorbing supporting structure under top impact	Area/(× 10^3^ kN·Hz)	410.43	222.23	128.69	128.35	152.09
	Area ratio/%	39.40	21.33	12.35	12.32	14.60
Traditional supporting structure under side impact	Area/(× 10^3^ kN·Hz)	721.18	269.83	236.63	198.23	224.27
	Area ratio/%	43.70	16.35	14.34	12.01	13.59
Energy absorbing supporting structure under side impact	Area/(× 10^3^ kN·Hz)	641.49	214.56	151.49	131.95	147.47
	Area ratio/%	49.85	16.67	11.77	10.25	11.46

## Analysis of the interaction between supporting structure and surrounding rock

### Synergistic system model

**Figure 10:**
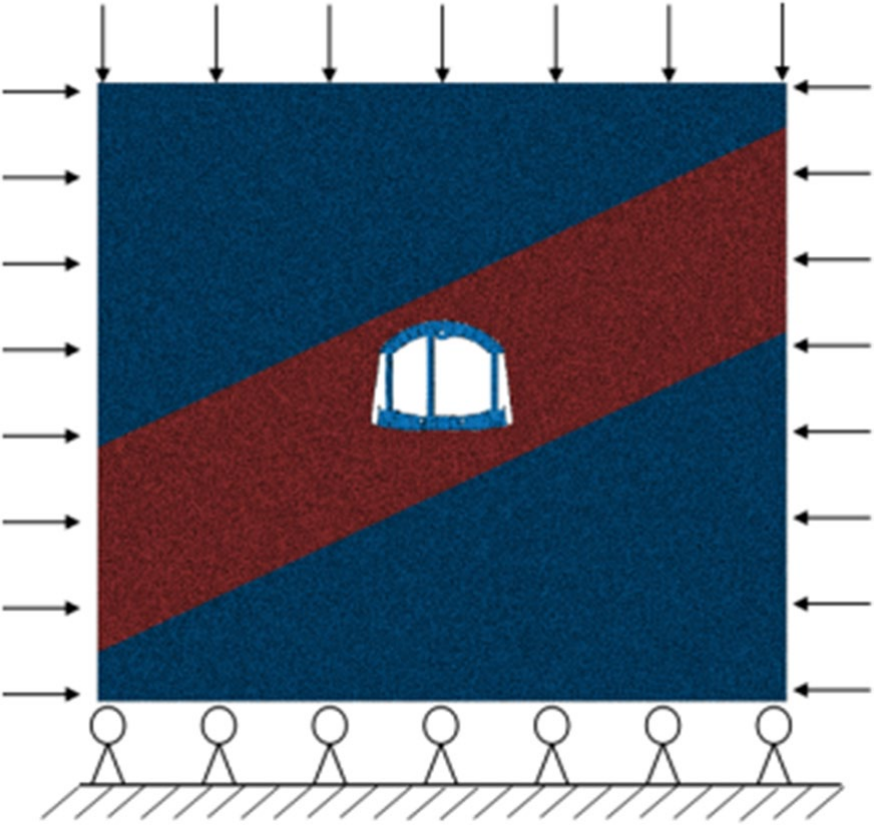
Synergistic system model.

According to the actual engineering conditions of the mine, the synergistic system model is established which is presented in Fig. 10. The surrounding rock is built based on the condition that the influence area of mining roadway is about 3–5 times of mining roadway area. Among them, the section of surrounding rock is a square with a side length of 22.5 m, a thickness of 0.5 m, a coal seam height of 7.5 m and a dip angle of 35°. Density of surrounding rock is 2000 kg/m^3^, gravity acceleration is 10 m/s^2^, and buried depth is 500 m. The vertical stress of surrounding rock is set to 10 MPa. To simulate the surrounding rock stress in different directions, the horizontal stress is set to 1.5 times of the vertical stress. The parameters of stratum are shown in Table 2. The mining roadway is built with 8-node 3D unit C3D8, with a grid size of 40 mm and a total of 205,856 elements.

**Table 2:** Mechanics parameters of rock stratum.

Material	Thickness (m)	Density (kg·m^3^)	Bulk modulus (GPa)	Shear modulus (GPa)	Cohesive strength (MPa)	Tensile strength (MPa)	Internal friction angle (°)
Fine sandstone	13.2	2530	13.4	7.6	1.4	11.55	37
Coal	7.5	1350	10.5	6.5	1.2	0.9	23
Coarse sandstone	1.8	2540	14.7	8.1	10.0	11.55	26

### Results of synergistic system model

**Figure 11:**
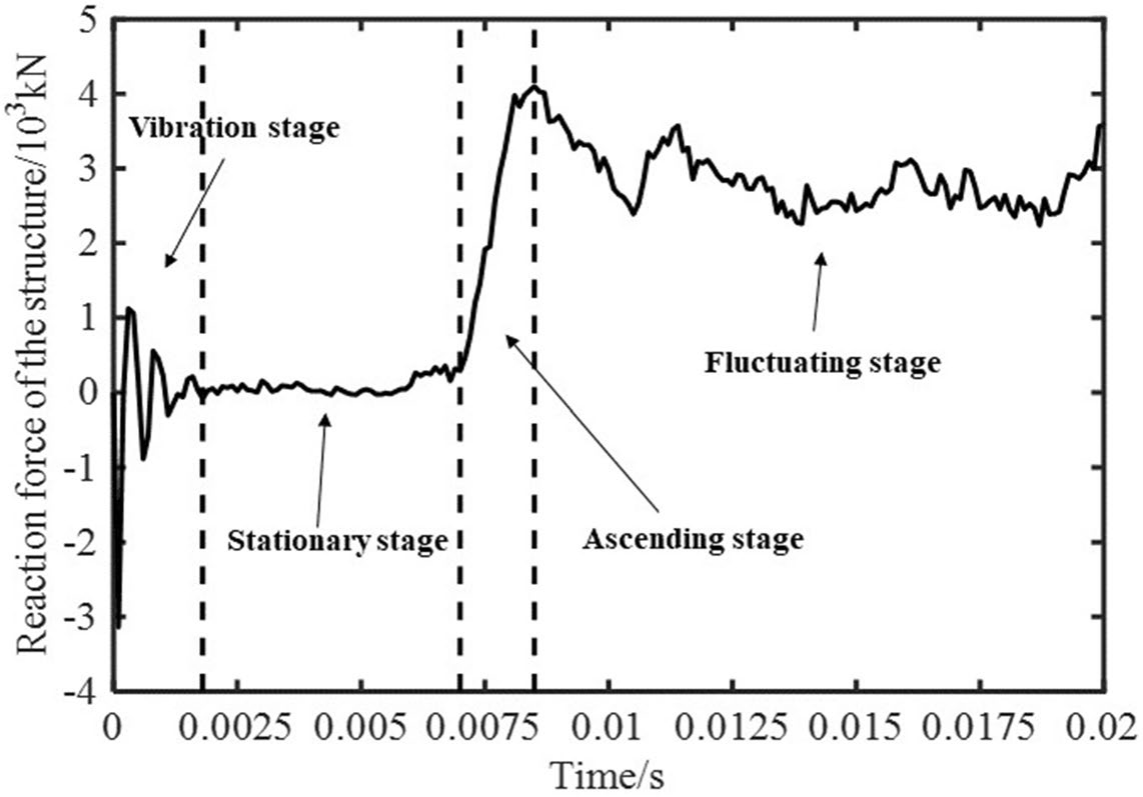
Reaction force curve of supporting structure in synergistic system under vertical impact condition.

Figure 11 presents the reaction force curve of supporting structure in synergistic system under vertical impact condition. The impact process in the figure is divided into four stages. Among them, the stage before 1.8 × 10^–3^ s is the vibration stage, during which the interaction force between the supporting structure and the surrounding rock is adjusted by high frequency vibration. The maximum reaction force at this stage is 3140 kN, and the frequency is about 2000 Hz. The range of 1.8 × 10^–3^–7 × 10^–3^ s is stationary stage. At this stage, the interaction force between supporting structure and surrounding rock fluctuates in a small range near zero, and the maximum fluctuation interval is only 93.7 kN. The curve with time in the range of 7 × 10^–3^–8.5 × 10^–3^ s is the ascending stage, during which the interaction force between supporting structure and surrounding rock rapidly increases to 4100 kN in a short time of 1.5 × 10^–3^ s. The curve with time in the range of 8.5 × 10^–3^–2 × 10^–2^ s is the fluctuating stage, and the interaction force between the supporting structure and surrounding rock is in a small fluctuation adjustment stage. The average reaction force of supporting structure in this process is 2860 kN. Comparing the reaction force in synergistic system with the force under top impact in “Results of energy-absorbing yielding anti-impact supporting structure model” section, it is found that the reaction force starts to rise rapidly after 0.007 s in the synergistic system, and the average reaction force of the fluctuation stage after rising is only 74.8% of the average reaction force under top impact. It can be seen that the interaction between the supporting structure and the surrounding rock delays the impact time and reduces the reaction force caused by the impact on the structure.

## Conclusion

1. Through the analysis on the properties of energy absorption device, it can be found that the deformation and yielding process under compression of it is divided into five stages, which are the first ascending stage, the first descending stage, the second ascending stage, the second descending stage and the third ascending stage.
2. Through the analysis on the properties of energy-absorbing yielding anti-impact supporting structure, the following conclusions can be drawn: Before the process of deformation and yielding of the energy absorption device reaches the third ascending section, the reaction force on supporting structure under impact can be effectively reduced. Regarding the fluctuation range of the reaction force, that of energy-absorbing supporting structure is smaller.
3. Compared with the energy-absorbing supporting structure without surrounding rock, the reaction force of that in synergistic system shows great differences: The value of the reaction force is lower and a stationary stage is added in the early stage of the reaction force curve. The result shows that the interaction between the supporting structure and surrounding rock delays the impact time and reduces the reaction force caused by the impact on the structure.

## Data Availability

All data, models, or code that support the findings of this study are available from the corresponding author upon reasonable request.
